# The Epidemiology of Pulmonary Nontuberculous Mycobacteria: Data from a General Hospital in Athens, Greece, 2007–2013

**DOI:** 10.1155/2014/894976

**Published:** 2014-06-10

**Authors:** Marios Panagiotou, Andriana I. Papaioannou, Konstantinos Kostikas, Maria Paraskeua, Ekaterini Velentza, Maria Kanellopoulou, Vasiliki Filaditaki, Napoleon Karagiannidis

**Affiliations:** ^1^2nd Respiratory Medicine Department, Sismanoglio-A. Fleming General Hospital of Attiki, Sismanogliou 1, 15126 Athens, Greece; ^2^2nd Respiratory Medicine Department, University of Athens Medical School, Attikon Hospital, Smolika 2, 16673 Athens, Greece; ^3^Department of Biopathology, Sismanoglio-A. Fleming General Hospital of Attiki, Sismanogliou 1, 15126 Athens, Greece

## Abstract

*Background*. The epidemiology of pulmonary nontuberculous mycobacteria (NTM) in Greece is largely unknown. *Objectives*. To determine the incidence and the demographic, microbiological, and clinical characteristics of patients with pulmonary NTM infection and pulmonary NTM disease. *Methods*. A retrospective review of the demographic, microbiological, and clinical characteristics of patients with NTM culture-positive respiratory specimens from January 2007 to May 2013. *Results*. A total of 120 patients were identified with at least one respiratory NTM isolate and 56 patients (46%) fulfilled the microbiological ATS/IDSA criteria for NTM disease. Of patients with adequate data, 16% fulfilled the complete ATS/IDSA criteria for NTM disease. The incidence of pulmonary NTM infection and disease was 18.9 and 8.8 per 100.000 inpatients and outpatients, respectively. The spectrum of NTM species was high (13 species) and predominated by *M. avium-intracellulare *complex (*M. avium* (13%), *M. intracellulare* (10%)), *M. gordonae* (14%), and *M. fortuitum* (12%). The ratio of isolation of NTM to *M. tuberculosis* in all hospitalized patients was 0.59. *Conclusions*. The first data on the epidemiology of pulmonary NTM in Athens, Greece, are presented. NTM infection is common in patients with chronic respiratory disease. However, only a significantly smaller proportion of patients fulfill the criteria for NTM disease.

## 1. Introduction

Nontuberculous mycobacterial (NTM) species are mycobacterial species other than those classified to the* Mycobacterium tuberculosis* complex (e.g.,* M. tuberculosis (Mtb)*,* M. bovis, M. africanum*, and* M. microti*) and* M. leprae* [1]. Despite being of the same family, NTM differ from those organisms that cause tuberculosis (TB) and leprosy in that they are widely dispersed in our environment, vary greatly in their ability to cause disease, and are not spread from person to person [2]. To date over 160 different species and subspecies of mycobacteria have been included in the List of Prokaryotic Names with Standing in Nomenclature (LPSN; http://www.bacterio.net) but the total number of mycobacterial species is constantly rising due to improved microbiological techniques for isolating NTM from clinical specimens and, more importantly, due to advances in molecular techniques for defining new species [1]. Accordingly, a spectrum of virulence has been identified ranging from primary pathogens such as* M. kansasii* that can cause disease in presumably healthy individuals and* M. avium* that is associated with preexisting lung disease or defects of cellular immunity to species such as* M. gordonae* that are rarely associated with disease [3].

Traditionally, pulmonary diseases have been reported to account for up to 94% of cases of NTM disease [4] and pulmonary NTM disease commonly occurs in the context of chronic lung disease, such as chronic obstructive pulmonary disease (COPD), bronchiectasis, cystic fibrosis (CF), pneumoconiosis, prior TB, and esophageal motility disorders [1]. Abnormal CF genotypes and *α*1-antitrypsin phenotypes may predispose some patients to NTM infection [1]. Pulmonary NTM disease also occurs in women without clearly recognized predisposing factors. Bronchiectasis and NTM infection, usually* M. avium-intracellulare* complex (MAC), often coexist, making causality difficult to determine [1]. Worryingly, certain MTN species, including MAC, are associated with high failure treatment rates and increased morbidity and mortality [5]. The prolonged treatment period, drug side effects and interactions, and possibly reinfection rather than relapse have been implicated in the treatment failure [5].

Defining the epidemiology of NTM is challenging for several reasons [6]. First, humans are thought to contract the infection directly from environmental sources. There has been no published report of direct or indirect patient-to-patient respiratory spread of NTM with the sole exception of an outbreak of respiratory* M. abscessus* disease in inpatient population with cystic fibrosis [7]. Second, exposure to the omnipresent MTM is likely extremely common. Third, NTM that colonize the respiratory tract can be isolated in respiratory samples in the absence of disease [1]. Lastly, in most regions of the world, NTM disease is not reportable to public health authorities; therefore, epidemiological and surveillance data are not readily available [6]. It is therefore not surprising that, until recently, there have been virtually no population-based data for America and only limited representative population-based data for Europe available that, as Winthrop et al. [8] eloquently state, firmly document those most basic questions of epidemiology: the “who, what, where, and how much?” [9].

Despite obstacles in the study of the epidemiology of pulmonary NTM, available evidence suggests that the prevalence of pulmonary NTM disease has increased dramatically globally over the past 3 decades [6]. It is also likely that this trend will continue to rise [10] and this is believed to be multifactorial. First, an increasing proportion of the population is aging or subject to some type of immunosuppression, supported by the increase in prevalence of immune-modulating comorbidities like diabetes mellitus and chronic obstructive pulmonary disease (COPD) and in immunosuppressive medication use [11]. Second, NTM are generally free-living organisms and are ubiquitous in the human environment. NTM are present in water, biofilms, soil, and aerosols and, importantly, are natural inhabitants of the piped water supply systems [10, 12, 13]. Thus, it is likely that humans are exposed to NTM on a daily basis [10]. Simultaneously, behavioral changes such as the rising use of swimming pools and hot tub baths may increase the likelihood of exposure to NTM [14]. Third, ongoing advances in methodology in the mycobacteriology laboratory have led to enhanced isolation and more rapid and accurate identification of NTM from clinical specimens [1]. A possible alternative explanation might be the development of cross-immunity between* Mtb* and certain types of NTM. Early studies have produced immunity to* Mtb* in animals by use of* M. kansasii* and MAC [15] and therefore it is felt that there is every reason to believe that this could occur in humans also [16]. If such cross-immunity does exist, the well-known decrease of TB worldwide [17] could have led to a decrease of immunity to NTM [6]. Finally increased awareness on behalf of the physicians might have led to more thorough investigation and follow-up of patients [18].

The aim of this hospital-based study was to determine the incidence of the isolation of NTM and the frequency of various NTM species. We also evaluated the clinical and demographic characteristics of patients with NTM and attempted to identify possible differences between patients colonized with and those who were actually diseased by NTM in terms of comorbidities and use of inhaled corticosteroids.

## 2. Materials and Methods

### 2.1. Setting and Data Collection

The study included consecutive adult in- and outpatients assessed at Sismanoglio-A. Fleming General Hospital of Attiki (SGH) from January 2007 through end of May 2013 from whom at least one biological sample was tested culture-positive for NTM. SGH is a 450-bed capacity hospital with a large number of outpatient clinic visits daily and the second largest tertiary referral hospital for patients with respiratory disease in Athens, Greece. SGH has a level II mycobacteriology laboratory with extensive experience in the field also was empowered to conduct limited-scale level III (reference-level) laboratory tasks.

Initial data were gathered from the database of the Department of Biopathology of SGH and included patient identification, species of the isolated NTM, isolation source, and patient demographics as described later. Multiple identical isolates from the same site during the same hospital episode in a single individual were counted as one patient entry. Subsequently, the medical records of these patients were reviewed with the aim of identifying relevant clinical characteristics as described later.

Ethics approval for this study was granted from the Institutional Review Board.

### 2.2. Specimen Processing

The clinical specimens were decontaminated using N-acetyl-L-cysteine-sodium hydroxide (NALC) in a Type 2 Biosafety Cabinet. All specimens were then inoculated into solid Löwenstein-Jensen (bioMerieux, Marcy, l' Etoile, France) and into 7H9 Middlebrook Broth Base 0.47% w/v (MGIT, Becton Dickinson, USA) media. Solid medium cultures were incubated in a 37°C incubator for 60 to 70 days and monitored every four days, whereas liquid cultures were incubated in an automated Bactec MGIT-960 (Becton Dickinson, USA) system for 45 days. Cultures exhibiting growth were subjected to light microscopy for the presence of acid-fast bacteria before being considered as positive. All positive cultures were subsequently analyzed by the GenoType Mycobacterium CM (Hain, LifeSciences, Germany) molecular genetic assay for identification of* Mtb* complex and 15 of the most common NTM species. Sporadically throughout the study period, positive cultures were analyzed with both GenoType Mycobacterium CM and GenoType Mycobacterium AS assays for identification of additional 15 less common NTM species. In this paper, NTM species identified neither by GenoType Mycobacterium CM nor by GenoType Mycobacterium AS (when used) assays are referred to as “unidentified NTM”.

### 2.3. Definition of Pulmonary NTM Disease

Patients were considered as having pulmonary NTM disease if they met the clinical, radiological, and microbiological characteristics as defined by the 2007 American Thoracic Society and Infectious Disease Society of America (ATS/IDSA) statement: Diagnosis, Treatment, and Prevention of Nontuberculous Mycobacterial Diseases [1].

### 2.4. Definition of Pulmonary NTM Infection (Colonization)

We defined NTM-infected (colonized) subjects as those who had at least one positive culture for NTM without fulfilling the complete diagnostic criteria and without any record of treatment for NTM disease.

### 2.5. Patient Characteristics

Demographic variables included age (at specimen's collection date), sex, ethnicity, and country of residence.

Clinical characteristics included the principal working diagnosis at the time of the specimen collection and the underlying medical conditions. The underlying conditions evaluated included chronic lung disease such as COPD, asthma, bronchiectasis, and old TB. Other conditions associated with immunosuppression including diabetes mellitus, HIV infection, autoimmune diseases, malignancy, chronic liver disease, and chronic renal disease were also logged. Finally we recorded the smoking status and long-term use of inhaled corticosteroids (ICS) and systemic corticosteroids (CS) prior to the diagnosis (7.5 mg or more of prednisone or equivalent daily for a period of two weeks or longer).

### 2.6. Statistical Analysis

Categorical variables are presented as* n* (%), whereas numerical variables are presented as mean ± SD. Comparisons between groups were performed using chi-square tests for categorical data and unpaired* t*-tests or Mann-Whitney* U* tests for normally distributed or skewed numerical data, respectively.

The incidence of pulmonary infection and disease caused by NTM for the duration of our study was calculated as the total number of patients with pulmonary NTM infection and disease divided by the total number of patients who attended SGH, including inpatients and outpatients.

All tests were two-tailed and *P* values <0.05 were considered statistically significant. Data were analyzed using SPSS 17.0 for Windows (SPSS Inc., Chicago, IL, USA).

## 3. Results

### 3.1. Study Population

**Figure 1:**
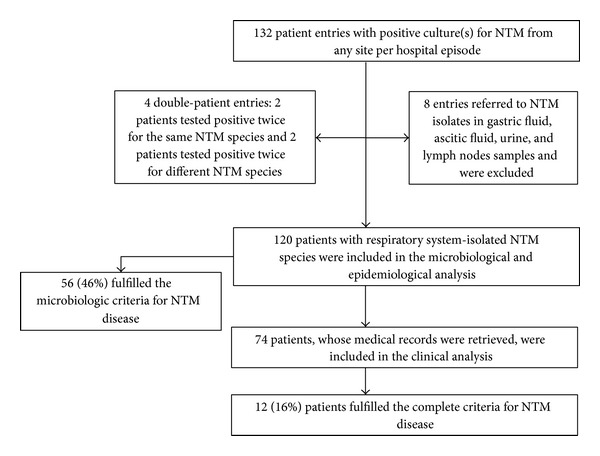
Flowchart of the study population.

A total of 132 patient entries with at least one positive culture for NTM from any site per hospital episode were identified. The majority of the identified subjects (95%) were inpatients in the respiratory medicine departments. Eight entries (6%) referred to NTM isolates in gastric fluid, ascitic fluid, urine, and lymph nodes samples and they were excluded from the analysis. Double entries were identified in four patients: two were tested positive twice for the same respiratory NTM species and two were tested positive twice for different respiratory species (all on separate hospital episodes). Therefore, we report on a total of 120 patients who had NTM species isolated from the respiratory system and they were included in the microbiological and epidemiological analysis. We were able to retrieve the medical records of 74 (61%) out of 120 patients; thus the analysis on clinical characteristics is relevant to only this subgroup of patients (Figure 1). The demographic and clinical characteristics of the patients are summarized in Table 1.

The study population consisted of 63% men, with a median age of 69.9 years, with the majority being born in Greece. The prevalence of NTM isolation increased with age, ranging from 0% in patients younger than 20 years to 82% in patients aged >60 years.

Out of the 74 patients included in the clinical analysis, 66% (*n* = 49) had a diagnosis of a chronic respiratory disease, including COPD (43%, *n* = 32), bronchiectasis (33%, *n* = 25), asthma (6%, *n* = 5), and cystic fibrosis (1%, *n* = 1). Seventeen percent (*n* = 13) of the subjects had a previous TB infection and 16% (*n* = 12) had lung cancer. Thirty-one percent (*n* = 23) were current users of ICS and 13% (*n* = 10) were current systemic CS users. Fifty-eight (*n* = 43) were current or ex-smokers. None of the patients was positive for HIV infection. Sixteen percent (*n* = 12) of patients fulfilled the complete criteria [1] for pulmonary NTM disease; all of them had an underlying chronic lung disease. However, the diagnosis of NTM disease was missed in 25% (*n* = 3) of them. No statistically significant differences were identified between colonized and diseased patients in terms of demographic and clinical characteristics.

### 3.2. Mycobacteriology Data

**Figure 2:**
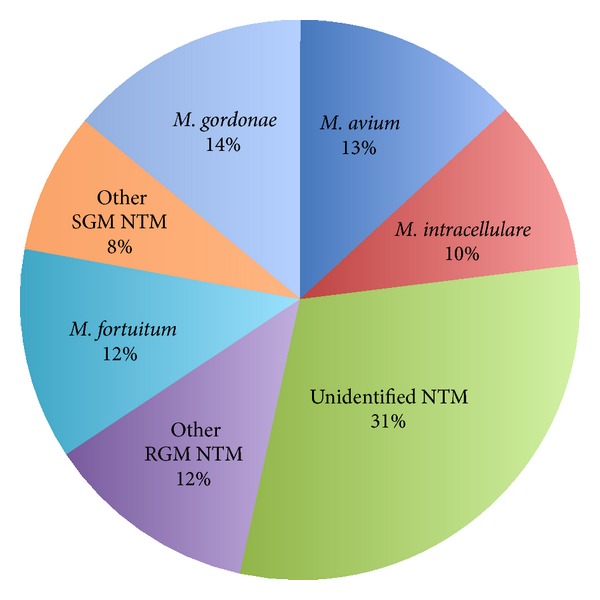
Diversity of isolated nontuberculous mycobacteria (NTM). RGM: rapidly growing, SGM: slowly growing.

One hundred and twenty-two NTM isolates were identified. Only ten percent (*n* = 13) of all respiratory specimens were AFB smear-positive, whereas, by definition, all were culture-positive. The spectrum of NTM species was high (13 species), the most common being the slowly growing* MAC* (*M. avium* (*n* = 16, 13%),* M. intracellulare* (*n* = 12, 10%)) and* M. gordonae* (*n* = 17, 14%) and the rapidly growing* M. fortuitum* (*n* = 15, 12%). Unidentified NTM species accounted for 30% (*n* = 37) of isolates (Table 2 and Figure 2). The pathogens accounted for the twelve cases of NTM disease including* M. avium* (*n* = 3),* M. intracellulare* (*n* = 3),* M. abscessus* (*n* = 1),* M. gordonae* (*n* = 1),* M. fortuitum* (*n* = 1),* M. xenopi* (*n* = 1), and unidentified NTM (*n* = 2). Fifty-six patients (46%) fulfilled the microbiological criteria of the ATS/IDSA for NTM disease.

In an attempt to identify the frequency of NTM isolates compared to that of* Mtb* we also extracted the number of isolates for* Mtb*. Within the study period, 225 patients were culture-positive for* Mtb*, rendering a ratio of NTM-to-*Mtb* isolation of 0.59. One patient (1%) was tested positive for* Mtb* and also fulfilled the microbiological criteria of the ATS/IDSA for NTM disease. He was put on standard anti-TB treatment.

### 3.3. Incidence of NTM Pulmonary Infection

During the 65-month study period, 138.951 inpatients and 492.845 outpatients where treated at SGH. Accordingly, the incidence of NTM pulmonary infection and disease for the study period was 18.9/100.000 and 8.8/100.000 patients, respectively.

## 4. Discussion

During the 65-month period of our study, a total of 120 patients were identified with at least one positive culture for respiratory NTM isolate and 56 patients fulfilled the microbiological criteria of the ATS/IDSA for NTM disease. The predominant NTM species were MAC (*M. avium* (13%),* M. intracellulare* (10%)),* M. gordonae* (14%), and* M. fortuitum* (12%). Of patients with adequate data, 16% fulfilled the complete clinical, radiological, and microbiological criteria of the ATS/IDSA for NTM disease. In approximately 25% of patients the diagnosis of NTM disease was missed. The incidence of pulmonary NTM infection and disease for the study period was 18.9 and 8.8 per 100.000 inpatients and outpatients, respectively.

To our knowledge, this is the first study on the epidemiology of NTM performed in Athens, Greece. Two additional studies on the incidence of NTM in Greece were found in the literature: one conducted in Larissa, central Greece [19], and one in Crete island [20]. Notably, all three are hospital-based studies, which highlights the lack of large-scaled epidemiological studies for NTM in Greece. Finally, data from the Greek National Reference Laboratory for Mycobacterium on NTM isolates in 2008 are reported elsewhere [21]. Also data on environmental sources of NTM in Greece are scarce although, in line with global evidence [10, 12, 13], unpublished data suggest that the municipal water systems are an important reservoir for infection [22]. Unfortunately, the only other study that has reported on the incidence of pulmonary NTM disease to date was based on different methodology (reporting a 3-year incidence rate of 0.7 per 100 000* general* population in central Greece for 2000–2003) [19]. The latter, along with the fact that NTM infection is not notifiable in Greece, does not allow any comparisons or conclusions to be drawn regarding trends and/or geographical variations in the epidemiology of pulmonary NTM in Greece.

In our study, 94% percent of the isolates stemmed from respiratory specimens. This is similar to other studies reporting that around 90% of all NTM isolates were of respiratory origin [4, 23, 24]. However, it is well established that isolation of NTM in microbiological samples most commonly represents simple exposure rather than disease. Therefore, laboratory-based studies tracking the incidence of NTM isolation in the population cannot distinguish between diseased and nondiseased persons [9]. However, access to patient clinical and radiographic records is laborious and not always feasible [25], and this represents a major obstacle for large-scale epidemiological studies of NTM disease. Thus, it is of no surprise that only few population-based studies attempted to look into the epidemiology of NTM disease outside the boundaries of the laboratory records. A national-scaled study that attempted to record the clinical significance of pulmonary NTM infections in New Zealand in 2004 by contacting the requesting clinician reported a specific incidence for pulmonary NTM disease of 1.17 per 100,000 population [26]. More recently, a population-based study evaluated the burden of hospitalization associated with pulmonary NTM infections in Germany in 2005–2011. The cases were identified using discharge diagnosis codes. The average annual age-adjusted rate was 0.91 hospitalization per 100,000 population [27]. Another population-based study in British Columbia, Canada from 2000–2006, combined laboratory data and data from the pharmacy department (but not patient records) to estimate the median incidence rates for all-NTM-colonized patients and all-NTM-treated patients being 4.7 and 1.6 per 1000 000 population, respectively [28].

As an alternative method for the epidemiological analysis of the NTM many laboratory-based studies relied upon the microbiological criteria of the ATS/IDSA case definition (validated positive predictive value 85% [8]) in order to estimate disease prevalence. This method assumes that for patients meeting the microbiological criteria, they have coexistent radiographic abnormalities and symptoms compatible with NTM disease. Although the accuracy of this approach is unknown, it is likely to be a reasonable assumption, as most patients undergoing bronchoscopic or sputum evaluation are doing so because of radiographic or symptomatic findings. One such study, which was carried out in all NTM isolates between 1987 and 2000, in the Southwest Region of Ireland reported a mean incidence of disease-causing NTM of as low as 0.4/100 000/years [29]. A population-based study conducted at Oregon, USA, for 2005-2006 reported the estimated annual pulmonary NTM disease prevalence to be 5.6/100,000 statewide but as high as 15.5/100,000 for those over 50 years of age [23]. Subsequently, the same research team undertook a new study to review the clinical records of a significant subset of these patients [8]. That was the first study to determine pulmonary NTM disease prevalence within a population and the first to systematically examine both the clinical and epidemiologic features of pulmonary NTM disease from a general population. They reported an upper limit 2-year prevalence estimate of 11.2/100,000 in the general population, which was—as one might have expected—lower than the prevalence of the microbiologically only defined NTM disease. Notably, the upper limit 2-year prevalence estimate in those at least 50 years old was 25.7/100,000, thus providing further evidence that NTM disease involves older population [8].

In line with aforementioned evidence, we report a higher number of patients who fulfilled the microbiological criteria of the ATS/IDSA for NTM disease compared to those who fulfilled the complete microbiological, radiological, and clinical criteria for NTM disease. Specifically, of patients with adequate data, only 37.5% of those who fulfilled the microbiological criteria of NTM infection also fulfilled the complete criteria for NTM disease. Another single-centered retrospective analysis reported similar proportion of patients (33%) who fulfilled all ATS/IDSA criteria for NTM disease out of the total number of patients with pulmonary NTM isolates [24].

In the present study, no statistically significant trends were observed in the yearly incidence of NTM from 2007 to 2013. However, a unifying finding in all the aforementioned studies was that the prevalence of pulmonary NTM infection and/or disease steadily increased during the study time period [24, 27, 29, 30]. For example, the annual number of all pulmonary NTM infection-associated hospitalization processes in Germany ranged from 665 in 2005 to 1,039 in 2011, with an average annual increase of 4.9% [27]. Also, the isolation prevalence of all MTN species in Ontario, Canada, was 9.1/100 000 in 1997, rising to 14.1/100 000 by 2003 and to 19/100,000 by 2007 (*P* < 0.0001) with a mean annual increase of about 8.5% [24, 31]. This increasing frequency was regarded as genuine rather than being based on physicians simply ordering more tests because the increase in isolation prevalence had not been accompanied by an increase in negative cultures [18]. In Taiwan, a three-year hospital-based study that retrospectively reviewed patient records reported a statistically significant rising incidence (per 100,000 inpatients and outpatients) of patients with pulmonary NTM disease (1.06 in 2005 and 2.00 in 2008) [32]. In USA, a comparison of skin test surveys revealed that, in 1999-2000, an estimated one in six persons demonstrated* M. intracellulare* sensitization compared to one in nine persons in 1971-1972 [33].

Virtually all studies from industrialized countries (including US, Canada, and Germany) that drew comparisons between the incidence and/or prevalence of pulmonary NTM disease and TB reported significantly higher rates for NTM [8, 24, 28, 34] or at least an increase in the ratio of NTM isolation prevalence to TB case prevalence increased during the study period [3, 24, 27]. However, incidence of TB may still outrange that of pulmonary NTM infection in most European countries [19, 27]. An analysis of data collected annually through the Greek national mandatory notification system for the period 2004–2010 shows that an average of 600 cases of TB are reported each year in Greece [35]. The estimated TB incidence is about 5 cases per 100,000 population, which ranks Greece as a low-burden country. Within our study period, the ratio of isolation of NTM to* Mtb* in all hospitalized patients was 0.59.

The distribution of NTM species worldwide varies by geographic region [21, 36]. MAC, which accounted for 23% of all NTM isolates and 50% of NTM pulmonary disease in our study, is the predominant pathogen in most regions worldwide [13, 21, 37–39]. Specifically, in a modern registry of 20182 patients, from 30 countries across six continents,* M. avium* predominated in North and South America and Europe, while* M. intracellulare* was most frequently isolated in South Africa and Australia [21].* M. kansasii* is relatively more common in the middle USA, Brazil, England and Wales, Eastern Europe and the metropolitan centres of Paris, London and Tokyo, and the Johannesburg region of South Africa;* M. xenopi* is more common in the northern USA, Ontario-Canada, UK, and some European countries including Hungary, Croatia, and Northern Italy;* M. malmoense* is common in UK and northern Europe but is uncommon in the USA and* M. simiae* is more common in arid regions of the southwestern USA, Cuba, and Israel [21, 30, 36]. Finally, rapidly growing mycobacteria (RGM), accounting for 10–20% of all NTM isolates worldwide in 2008, proved more prevalent in East Asia [21]. Interestingly, RGM made up 25% of all NTM isolates in our study and they were also the prevalent NTM species (46%) in Greece in 2008 [21]. Accordingly, it is possible that regional variations in environmental conditions may favor differences in the predominant NTM populations in the water and soil reservoirs to which susceptible patients are exposed [36]. It should be mentioned that all the identified NTM isolates were considered to be clinically significant in our study.* M. gordonae*, which is usually regarded as a nonpathogenic commensal, was also considered as ample evidence suggesting that it is still capable of causing clinically significant disease in both immunocompetent and immunosuppressed individuals [40, 41].

In terms of gender distribution of pulmonary NTM disease, there has been a gradual shift since early epidemiological data [1, 36]. Although reports from 50 years ago described lung disease most commonly in older smoking male with emphysema, in today's clinics, approximately 80% of patients with NTM disease are middle aged or elderly females with midlung bronchiectasis and other pulmonary abnormalities [36]. In the population-based Oregon, USA, study, females accounted for 60.5% of the pulmonary MAC disease, with a rate of 5.7 cases per 100,000 persons, compared with 3.7 cases per 100,000 persons among males [23]. Also, the New Zealand national-scaled study found the majority (79%) of cases of NTM disease to be females [26]. In recent studies, male sex has been associated with an elevated risk of sensitization (but not isolation) to NTM species [33]. Only one modern study reported moderately increased incidence of (microbiologically only confirmed) NTM disease in males and identified male gender as a risk factor for NTM disease in the Saudi Arabia population [42]. Although the increasing rates of smoking in women may partially account for this shift, it does not explain the also raising frequency of pulmonary NTM in nonsmoker females [36]. Notably, our data showing that NTM-diseased men outnumbered women by far (63% men and 37% women) are not in accordance with this gender shift in the epidemiology of pulmonary NTM. Similarly, the other studies on Greek populations reported that the incidence of pulmonary NTM in men was either higher [19] or equal [20] to that of women. This discordance with the international data is not well understood and it may well be due to the small size of the studies.

In our study, no statistically significant differences were identified between colonized and diseased patients in terms of demographics and other well-documented risk factors for pulmonary NTM disease, including chronic lung disease, nonpulmonary comorbidities, immunosuppression [1], and the use of corticosteroids [43]. This probably reflects the small population size rather than diversion of our population from the worldwide situation. Of interest is also the fact that approximately 30% of diseased patients had no identified coexisting conditions. Similar rates of absence of coexisting conditions among patients with pulmonary NTM disease have been reported in other recent studies [30], which might reflect a shift in the epidemiology of NTM disease in current years.

Finally, we identified a clinically significant minority (25%) of patients who fulfilled the full criteria for pulmonary NTM in whom the diagnosis was missed during their hospital admission. This group of patients did fulfill the complete criteria for NTM disease but, since the microbiological confirmation was not available before their discharge day, they were treated and discharged under the diagnosis of nonspecific lower respiratory tract infection. It is unknown whether they were tracked at a later stage and put on treatment for NTM disease. Indeed, the microbiology cultures for NTM may take prolonged time (up to several days or weeks) to turn positive. By that time, many patients have been discharged from the hospital and may later not attend their follow-up to system or they may simply be lost in the system. Also, physicians are often reassured by the negativity of fast acid smear in that they will not chase the culture results vigorously and in timely fashion. In our study, culture positivity of the specimens was associated with AFB smear positivity in only 10% of the cases. Consequently, patients may lose their opportunity to be diagnosed with pulmonary NTM disease and receive appropriate treatment. Nevertheless, this finding suggests that NTM disease might be underdiagnosed and thus contributes to the universally reported wide gap in the percentage of the patients with NTM infection and disease.

One study limitation was the fact that only 61% of patient records were retrieved and evaluated. This is of course a universal issue in retrospective record-based studies [8] but it still decreased our study power. Additionally, the population seen in our hospital is not representative of the wider population. Our hospital is considered one of the major respiratory hospitals in Athens; therefore, our patient sample is predominated by those with respiratory disease who, in turn, are more likely to harbor a NTM infection and/or disease. Additionally, our study design allowed for the estimation of the incidence of the* hospital* rather than the* general* population of pulmonary NTM. The latter was not feasible due to this being a single-center study and also because the catchment area of our center extends well beyond the city of Athens, thus serving a wider and difficult-to-estimate population from Greek islands and mainland Greece. Finally, the high incidence of unidentified NTM species in our study may be due to the use of a single molecular genotyping assay (GenoType Mycobacterium CM) that allows the identification of 15 of the most common NTM species. GenoType Mycobacterium AS assay, which allows the identification of 15 additional (although less common) NTM species, was employed only sporadically throughout the study period and also in-house diagnostic assays for NTM were not available. Undoubtedly, the use of more powerful assays would have increased the diversity of NTM isolates.

## 5. Conclusions

The first data on the epidemiology of NTM in Athens, the capital city of Greece, are presented from the database of a tertiary referral hospital for patients with respiratory disease. From 2007 to 2013, 120 respiratory isolates were identified mostly from patients with chronic respiratory disease. However, only a smaller proportion of patients fulfill the criteria for disease. It is not clear whether the latter truly reflects a low penetration of the disease or underdiagnosis and/or methodological issues. In this study, the diagnosis of pulmonary NTM disease was missed in a clinically significant minority of patients. Increased awareness on behalf of the physicians is required regarding the significant morbidity and mortality of the untreated NTM disease. Moreover full application of the validated clinical, radiological, and microbiological guidelines is imperative in order to correctly identify the cases of NTM disease. This study aspires to increase physicians' insight into the challenges in the management of patients with potential NTM disease and stimulate further and larger-scale research for better determination of the epidemiology of NTM in Greece and worldwide.

## Figures and Tables

**(a):** 

Gender	
Females	76 (63%)
Males	44 (37%)
Total age (years)	69.9 ± 15.4
Age group	
<20 years	0 (0%)
20–40 years	10 (8%)
40–60 years	12 (10%)
>60 years	98 (82%)
Ethnicity	
Greek	110 (92%)
Others (including Middle East, East Europe, and Balkans)	10 (8%)
Residence	
Greece	120 (100%)

**(b):** 

	All	Colonized (*n* = 62)	Diseased (*n* = 12)	*P* value
Chronic lung disease				
COPD	32 (43%)	26 (42%)	6 (50%)	0.606
Bronchiectasis	25 (33%)	22 (35%)	3 (25%)	0.635
Asthma	5 (6%)	4 (6.4%)	1 (8.3%)	0.892
Cystic fibrosis	1 (1.3%)	1 (1.6%)	0 (0%)	0.683
Old TB	13 (17%)	8 (13%)	5 (41%)	0.030
Use of CS				
Inhaled CS	23 (32%)	20 (32%)	3 (25%)	0.528
Systemic CS	10 (14%)	10 (16%)	0 (0%)	0.140
Smoking habit				
Current or ex-smokers	44 (59%)	37 (59%)	7 (58%)	0.221
Never being smokers	30 (41%)	25 (40%)	5 (42%)	0.223
Others				
HIV	0	0	0	N/A
Autoimmune disease	1 (1.3%)	1 (1.6%)	0 (0%)	0.683

Data are presented as mean ± standard deviation (SD) for numerical variables or as number (%) for categorical variables.

COPD: chronic obstructive pulmonary disease, HIV: human immunodeficiency virus, and CS: corticosteroid.

**Table 2:** NTM isolates from all sites.

	2007	2008	2009	2010	2011	2012	2013	Total number (% of total)
Rapidly growing NTM								
* M. fortuitum *	6	2	6	0	1	0	0	15 (12.2)
* M. peregrinum *	2	1	1	0	2	0	0	6 (4.9)
* M. chelonae *	2	0	0	0	0	1	0	3 (2.4)
* M. abscessus *	0	0	0	0	1	0	1	2 (1.6)
* M. smegmatis *	0	1	0	1	0	0	0	2 (1.6)
* M. mucogenicum *	0	1	0	0	0	0	0	1 (0.8)
* M. fortuitum mageritense *	0	0	1	0	0	0	0	1 (0.8)
Slowly growing NTM								
* M. gordonae *	2	2	0	3	5	2	3	17 (13.9)
* M. avium *	1	3	2	2	5	3	0	16 (13.1)
* M. intracellulare *	2	1	2	2	2	3	0	12 (9.8)
* M. lentiflavum *	1	0	3	1	0	1	0	6 (4.9)
* M. xenopi *	2	0	1	0	0	0	0	3 (2.4)
* M. scrofulaceum *	1	0	0	0	0	0	0	1 (0.8)
Unidentified NTM	7	3	9	4	6	6	2	37 (30.3)
Total per year	**26**	**14**	**25**	**13**	**22**	**16**	**6**	**122 (100.0)**
